# Evaluation of Polyvinyl Alcohol/Pectin-Based Hydrogel Disks as Extraction Phase for Determination of Steroidal Hormones in Aqueous Samples by GC-MS/MS

**DOI:** 10.3390/molecules24010040

**Published:** 2018-12-22

**Authors:** Naiara M. F. M. Sampaio, Natara D. B. Castilhos, Bruno C. da Silva, Izabel C. Riegel-Vidotti, Bruno J. G. Silva

**Affiliations:** Department of Chemistry, Federal University of Paraná, Curitiba/PR 81530-900, Brazil; naiaramfms@gmail.com (N.M.F.M.S.); nataraduane@gmail.com (N.D.B.C.); brunosvik@gmail.com (B.C.d.S.); iriegel@gmail.com (I.C.R.-V.)

**Keywords:** gas chromatography, hydrogel, hormones, pectin, polyvinyl alcohol, sample preparation

## Abstract

A new extraction phase based on hydrogel disks of polyvinyl alcohol (PVOH) and pectin was proposed, characterized and evaluated for the extraction of six steroidal hormones (estriol, estrone, 17β-estradiol, 17α-ethinylestradiol, progesterone, and testosterone) in aqueous samples with subsequent determination by gas chromatography-tandem mass spectrometry (GC-MS/MS) after the derivatization procedure. The developed extraction procedure was based on the solid phase extraction (SPE) technique, but employed hydrogel as the sorbent phase. The effects of several parameters, including the amount and composition of the sorbent phase, pH, sample volume, flow rate, and gel swelling over the extraction efficiency, were evaluated. Gels with lower swelling indexes and larger amounts of sorbent ensured higher extraction yields of analytes. The main benefits of using the PVOH/pectin-based hydrogel as the extraction phase are the ease of synthesis, low-cost preparation, and the possibility of reusing the extraction disks. Limits of quantification of 0.5 μg L^−1^ for estrone and 17β-estradiol, and 1 μg L^−1^ for testosterone, 17α-ethinylestradiol, progesterone, and estriol were obtained. Accuracy values ranged from 80% to 110%, while the inter-assay precision ranged from 0.23% to 22.2% and the intra-assay from 0.55% to 12.3%. Since the sorbent phase has an amphiphilic character, the use of hydrogels is promising for the extraction of medium-to-high polarity compounds.

## 1. Introduction

Solid phase extraction (SPE) is the most popular sample preparation technique for analyte concentration and removal of matrix interferents, with several devices (cartridges and disks) and sorbent phases developed [1,2]. However, commercial phases, such as octadecylsilane (C18), have several disadvantages, including poor selectivity that leads to co-extraction of interferents, and difficulty in extracting polar compounds from aqueous matrices, since they present hydrophobic character. 

To overcome these problems, there are commercially available extraction phases capable of acting in a wider range of polarity, such as Oasis HLB^®^ and Strata-X^®^. In these cases, the retention mechanisms of the analytes occur through hydrophobic interactions, π-π interactions, and hydrogen bonding. Even so, SPE cartridges are expensive, especially those with sorbent phases with a hydrophilic–hydrophobic balance. Moreover, these devices are disposable and usually used for a single application [2].

Significant efforts have recently been made to develop new materials with high sorption capacities, selectiveness, stability (longer lifetimes) and low costs [3]. For the past 50 years, hydrogels have been applied in the medical and pharmaceutical fields for the development of artificial organs [4], tissue engineering [5], and controlled drug delivery systems [6]. Nevertheless, the use of hydrogels as sorbent phases has recently drawn attention, mainly because of their structures formed by highly hydrophilic polymeric networks that are capable of absorbing and retaining large amounts of water without dissolving [7]. The possibility of modulating the chemical and physical-chemical properties of these materials enables the production and use of more selective phases. 

Despite these attractive characteristics, the use of hydrogels in developing new extraction phases only started within the past decade and is still scarcely explored. In the literature, few works can be found that employ hydrogels for extracting organochlorine pesticides [8] or organic contaminants [9] from water samples using a polymer-coated hollow fiber microextraction technique. Hydrogels have also been associated with others materials, such as chitosan for recognition and separation of albumin bovine serum [10], zirconia nanoparticle-decorated calcium alginate hydrogel fibers [11], and acrylated composite hydrogels [12] for the extraction of organophosphorus pesticides and methyl blue, respectively. In these applications, the interaction between the analytes and the extraction phase occurs on the material surface (hollow fiber and dispersive extractions). Until now, no work has been carried out to explore the interactions that occur inside the hydrogel, promoted by the permeation of the sample through the gel phase, as occurs in SPE-packed phases (cartridges or disks).

In the present work, a new extraction phase is proposed based on disks of hydrogel, which is an innovative material for extraction purposes and presents some advantages over the solid sorbent phases employed in SPE, since gels have rheological properties that are intermediate between that of solid and liquid materials. This approach consists of the following three steps: (1) gel hydration, (2) sample percolation, and (3) elution of the analytes. In this case, the extraction process does not require conditioning of the extraction phase with an organic solvent and allows for the use of high flow rates, thus reducing the time for sample preparation.

Highlighting the potential of hydrogel-based materials as sorbent phases, hydrogels of polyvinyl alcohol (PVOH) and the biopolymer pectin were developed. PVOH is a semi-crystalline hydrophilic synthetic polymer produced by the hydrolysis of polyvinyl acetate (PVA). The PVOH structure contains several –OH groups and is, therefore, a highly hydrophilic and water-soluble polymer that exhibits excellent mechanical properties, chemical stability and the ability to form films [13]. Pectin is a polysaccharide extracted from plants and is mainly composed of α(1-4)-linked galacturonic acid (GalA) units containing varying amounts of methyl ester substituents (methoxylation degree), depending on the pectin’s origin and purification method [6,14]. Moreover, the presence of hydrophilic sites, such as –OH and/or –COOH in the PVOH and pectin, is expected to promote the extraction of compounds of high polarity.

The proposed PVOH/pectin-based hydrogel disk as an extraction device was characterized and applied for the extraction of medium-to-high polarity organic compounds from aqueous samples. For this purpose, six steroidal hormones were selected as the analytes (estriol (E3), estrone (E1), 17β-estradiol (E2), 17α-ethinylestradiol (EE2), progesterone (PRO), and testosterone (TES)) and the determination was carried out using gas chromatography-tandem mass spectrometry (GC-MS/MS). 

## 2. Results and Discussion

### 2.1. Physical-Chemical Characterization of the Hydrogel

The hydrogel disk performance was characterized in relation to several parameters, including mass, composition, morphological aspects, and swelling index (SI) of the hydrogel. Hydrogel disks prepared only with PVOH (>99% hydrolyzed) did not allow any water permeation through the gel. As highly hydrolyzed PVOH was employed, a gel with a high degree of reticular closure was obtained, thus hindering the permeation of water through the hydrogel. 

The addition of pectin into the hydrogel increased the amphiphilic character of the sorbent phase, due to presence of methyl ester groups in the pectin structure. This assured high extraction efficiencies for all hormones, especially for progesterone, which has the highest octanol/water partition coefficient (log Ko/w 3.87 [15]) among the analytes. The addition of this natural polymer to PVOH proved to be advantageous since it allowed for permeation of the samples and extraction of the analytes and did not affect the excellent mechanical properties of PVOH.

**Figure 1:**
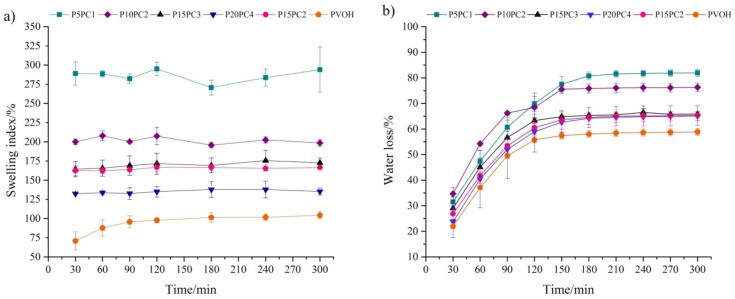
(**a**) Swelling index (SI) (wt.%) and (**b**) water loss (wt.%) of the hydrogels (*n* = 3) at 25 °C.

The swelling degree of hydrogels is dependent on several factors, such as the chemical structure, molar mass, composition and, degree of crosslinking of the polymer matrix [16]. Figure 1a shows the swelling behavior of P5PC1, P10PC2, P15PC3, P20PC4, P15PC2, and pure PVOH gels crosslinked with citric acid. For the differentiation of each gel composition studied, the acronym PxPCy was adopted, where x corresponds to the initial concentration (%, *w*/*v*) of PVOH dispersion and y the initial concentration (%, *w*/*v*) of pectin dispersion. In Figure 1a, it can be observed that for the hydrogels containing pectin, the equilibrium swelling was reached within the first 30 min, whereas for pure PVOH, 90 min was required. In addition, the presence of pectin resulted in higher water uptake, while for PVOH the SI was 104.3%. Gels containing pectin presented values ranging from 277.2% (P5PC1) to 135.2% (P20PC4). P5PC1 and P10PC2 disks absorbed the largest amounts of water and obtained the lowest extraction efficiencies in comparison to the others. This fact indicates that there is an optimal SI that favors the interactions between the analytes and the sorbent phase.

The swelling process of gels is governed by the mobility of the polymeric network, which defines the distance between the chains and, consequently, the volume available for the permeation of the solvent and the transport of solutes [17]. Therefore, for hydrogels that absorb larger volumes of water, the distance between the chains of the polymeric network is greater, which impairs the retention of analytes by the sorbent phase. 

The water loss process was studied at room temperature (25 °C) and under refrigeration (4 °C). However, since both temperatures had practically the same behavior, except that the mass equilibrium was reached faster at 25 °C, only the results for room temperature are presented as a graph (Figure 1b). The hydrogels presented similar behavior regarding the water loss process, with the highest rate being ~80% for P5PC1 and the lower rate being ~50% for PVOH. For the gels prepared with a lower polymer mass, the diffusion process of the solvent in and out of the polymer network was more pronounced. Higher diffusion rates did not favor the extraction of the hormones, since these compounds have high affinity with the aqueous matrix, and a shorter contact time of the sample with the sorbent phase may have contributed to the low extraction efficiencies of the P5PC1 and P10PC2 gels.

**Figure 2:**
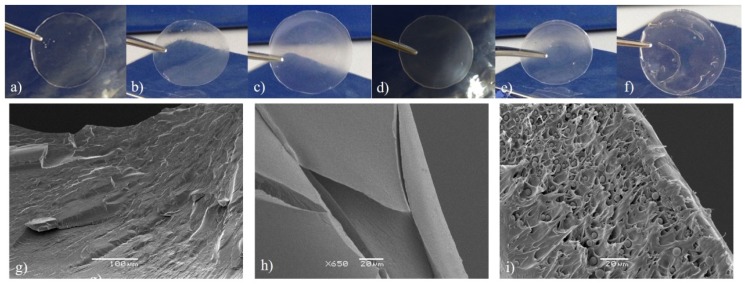
Hydrogel disk pictures of (**a**) pure polyvinyl alcohol (PVOH); (**b**) P5PC1; (**c**) P10PC2; (**d**) P15PC2; (**e**) P15PC3 and (**f**) P20PC4. Scanning electron microscope (SEM) images of (**g**) pure PVOH 250×; (**h**) pure pectin 650x and (**i**) PVOH/pectin extraction disk (P15PC2) 750×.

The morphological aspects of pure PVOH, pure pectin and PVOH/pectin phases are shown with photographs in Figure 2a–f and scanning electron microscopy (SEM) images in Figure 2g–i. It can be noted in Figure 2i that the presence of spherical particles corresponds to the pectin phase. The spherical shape of the pectin particles indicates that the two polymeric phases are immiscible. The addition of pectin into the hydrogels enhanced the PVOH surface area, leaving PVOH functional groups available to interact with the analytes and also assigning a more amphiphilic character to the sorbent phase. The average thickness of the hydrogel disks was between 150 and 200 μm.

**Figure 3:**
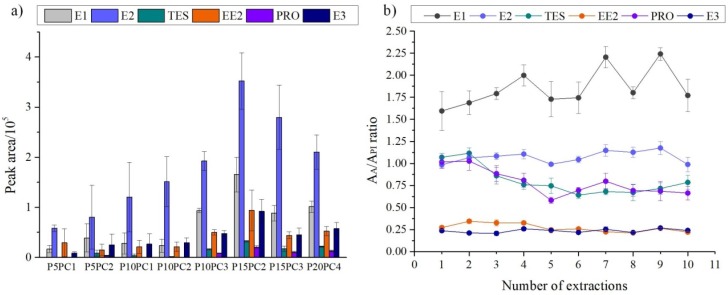
(**a**) Peak areas (*n* = 3) of the hormones (1 μg mL^−1^) extracted from ultrapure water according to the hydrogel disk used, where P = PVOH and PC = pectin. (**b**) AA/AIS ratios of 10 consecutive extractions with the same hydrogel disk (*n* = 3) for each hormone. AA: analyte peak area; AIS: internal standard peak area.

The hydrogel-SPE efficiency (Figure 3a) was evaluated as a function of the total amount of sorbent phase. PVOH/pectin hydrogel disks were prepared, maintaining a ratio of 5:1, but increasing the polymer mass and resulting in extraction phases with different mass: P5PC1 (19.03 mg), P10PC2 (44.62 mg), P15PC3 (52.67 mg) and P20PC4 (63.86 mg). Different ratios of the initial concentrations of the dispersions were also used as follows: 3.3:1 (P10PC3), 7.5:1 (P15PC2), and 10:1 (P10PC1). This variation in the ratio of PVOH to pectin was studied since the addition of pectin can alter the polarity of the extraction phase, being able to interfere in the retention mechanisms of the analytes. Another reason relates to the swelling index that depends on the composition of the gel and has an influence on the extraction efficiency.

It was found that higher amounts of sorbent led to an increase in the extraction efficiency up to P15PC2. This fact may be linked to an increase in the amount of pores, which makes analyte desorption more difficult. In addition, the P20PC4 gel (Figure 2f) was less uniform than the others due to the high viscosity of the initial mixture (PVOH at 20% and pectin at 4% *w*/*v*). Another important aspect was that the use of low amounts of polymers (P5PC1 and P10PC2, Figure 2b,c, respectively) produced very moldable gels that folded inside the holder, which could cause the sample to percolate by preferential flow paths. The highest extraction efficiency was achieved for the P15PC2 hydrogel disk (Figure 2d), which was adopted as the sorbent phase for the hydrogel-SPE and employed in the subsequent studies. 

### 2.2. Optimization of Hydrogel-Solid Phase Extraction (SPE) Conditions

A multivariate study was carried out through a 2^3^ full factorial design using the P15PC2 disk as the extraction phase. Higher extraction efficiency was obtained when the pH, sample volume and flow rate conditions were 7.5, 100 mL and 4 mL min^−1^, respectively. Pareto charts (Figure S1—Supplementary Material) shows that only E1, E2, EE2 and E3 presented significant main and interaction effects. 

#### 2.2.1. Effect of pH

The pH was studied regarding the stability of the gel and the extraction process. In the pH range from 3.0 to 9.0, all the sorbent disks were stable. Therefore, for the extraction process, pH values of 4.5, 6.0 and 7.5 were employed. The pH effect was not relevant for any hormone; however, second and third-order effects could be observed (Figure S1—Supplementary Material). In this pH range, all the hormones are in their non-ionized form (pKa between 10.2 and 15.1 [18]), which guarantees a higher affinity of the analytes with the sorbent phase. 

Therefore, the pH will also influence the degree of opening or closing of the polymeric network, since the degree of swelling of the hydrogel phase may be altered due to ionization or dissociation of functional groups. For example, when raising the pH, the OH^-^ ions hydrolyze the remaining acetates (pKa = 4.76) of PVOH. Then, charges present in the polymer chain, due to the ionized groups, repel each other and the degree of swelling increases [19]. By opening the network, due to the water absorption, it is assumed that there will be an increase in the rate of solvent diffusion into the network. It was observed that higher diffusion rates had a negative effect on the extraction efficiency (in terms of peak area), because of the insufficient contact time of the hormones with the hydrogel, which is a preferable closer network; therefore, higher pH values led to an improvement in the extraction rates, which was due to an increase in the ionic strength, leading to neutralization of the charges

#### 2.2.2. Effect of Sample Volume and Flow Rate on the Extraction Efficiency

Increasing the sample volume from 100 to 200 mL did not promote an increase in the response. Instead for most of the analytes, the AA/AIS ratio was lower when the sample volume was 200 mL. Higher sample volumes can lead to a higher enrichment of analytes; however, they can also cause desorption of compounds from the sorbent phase, even in the extraction step, especially when these compounds are polar and have great affinity with water. 

The flow rate effect was significant for the four hormones (E1, E2, EE2, and E3) and had the greatest influence over the extraction efficiency of the PVOH/pectin hydrogel. As the compounds studied have medium to high polarity, a longer contact time of the sample with the hydrogel, due to the use of lower flow rates (2 mL min^−1^), was not beneficial to the extraction process because it promoted the elution of hormones by the sample. Thus, higher flow rate (4 mL min^−1^) was selected.

#### 2.2.3. Optimization of Desorption Volume

The desorption step was studied in order to use the lowest volume of organic solvent possible, as well as evaluating the possibility of a memory effect in consecutive extractions using the same extraction disk. For this purpose, the use of six elution steps (1.0 mL of methanol for each one) was evaluated in triplicate. The number of elutions was determined by establishing that the accumulated peak area should be higher than 90%, which was reached with a total of five elutions.

The proposed method required 5 mL of organic solvent for the elution step; meanwhile, the literature usually reports the use of 8 to 15 mL [20,21,22,23] of organic solvents when employing cartridges in SPE; for commercial disks, this amount is even higher and can reach 20 mL [23,24]. Therefore, hydrogel-SPE is an interesting approach because it does not require conditioning of the extraction phase with organic solvents and uses smaller amounts of elution solvent, which makes the method environmentally friendly and reduces time spent in concentrating the eluate.

### 2.3. Quantitative Parameters and Analytical Comparison with other Methods

From the beginning of this study, one of the main characteristics for the hydrogel extraction phase was that it could be reusable, which would guarantee not only commercial appeal to this new material, but also greater ease and acceptance of the new phases based on hydrogel for the SPE technique. Moreover, the reuse of the hydrogel disk also guarantees robustness of the proposed extraction phase. For all hormones, there was a difference of up to 20% in the standard deviation between the results of one extraction and another. E1, E2, and E3 had a deviation of only 10% (Figure 3b). These results show that it is possible to reuse the hydrogel extraction disks, which is an advantage in relation to commercial sorbent phases employed in SPE, where reuse is normally not feasible.

**Figure 4:**
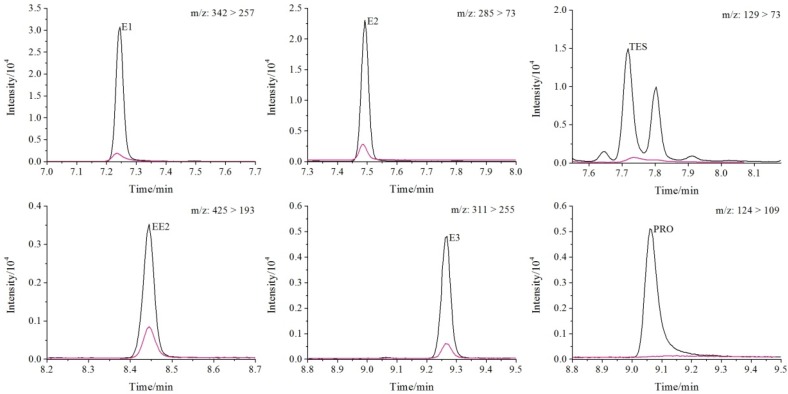
Chromatograms (multiple reaction monitoring, MRM) for a standard solution of hormones (500 µg L^−1^) (pink) and ultrapure water spiked with hormones (500 µg L^−1^) after extraction with polyvinyl alcohol (PVOH)/pectin disk (P15PC2) at optimized conditions (black).

Chromatograms of the standard solutions (pink line) and the application of P15PC2 hydrogel as the extraction phase for SPE (black line) for steroidal hormones can be seen in Figure 4. Enrichment factors of 500-fold were obtained based on the sample volume (100 mL) and the final volume of the extract (200 µL). Limits of quantification (LOQ) were estimated using the lowest point of the analytical curve with a relative standard deviation (RSD) less than 20%. The proposed method presented a linear relationship from the LOQ to 100 μg L^−1^ with all correlation coefficients higher than 0.99. For E1 and E2, the LOQ concentration was 0.5 μg L^−1^, while for TES, EE2, PRO, and E3, the LOQ value was 1.0 μg L ^−1^. The RSD (*n* = 3) values for the LOQ were lower than 12% and the accuracy was between 85.1% and 107.3% for all analytes. The limit of detection (LOD) ranged from 0.15 (E1, E2) to 0.30 μg mL^−1^ (TES, EE2, PRO, and E3), which was based on a signal-to-noise ratio 3.

In our study, the LOQ values were higher than those described in the literature using commercial SPE phases for analysis of hormones in environmental samples [21,23,25,26]. However, take into consideration the low thickness of the developed hydrogel disks (around 150 μm), we believe that the results are promising, especially in terms of precision, accuracy, low cost, and robustness. Since the thickness of the commercial SPE disk is about 500 μm, some modifications to the proposed device can be performed to achieve a lower LOQ. In future studies, for example, two strategies will be evaluated: i) increasing the diameter or thickness of the hydrogel-based disks, leading to a larger mass, and obtaining higher enrichment factors due to the increase in sample volume and ii) using the sandwich configuration, where more than one disk is placed inside the device in order to improve the detectability of the technique. In addition, the possibility of modulating the hydrogel disk by varying its chemical constitution can be evaluated for a group of target compounds, which may guarantee greater detectability of the analytical method.

Two concentration levels were evaluated for the intra (40.0 and 80.0 μg L^−1^) and inter-assay (50.0 and 100.0 μg L^−1^) precision experiments (performed in triplicate). The RSD values were lower than 13%, except for the PRO inter-assay at 50 μg L^−1^ (22%). The hydrogel-SPE technique proved to be reproducible for the extraction of hormones from aqueous samples since the RSD value was less than 13%, using different extraction disks for each precision assay. In addition, accuracy results of 80% to 110% demonstrated that it was possible to use the proposed hydrogel as the extraction phase to quantify organic compounds of medium to high polarity present in aqueous matrices. Table 1 summarizes these results. 

In this work, precision and accuracy were similar to those obtained in previous studies. For example, Migowska et al. [27] extracted E1, E2, EE2, and E3 from surface water with an Oasis HLB cartridge with determination by GC-MS, where the RSD values for intra- and inter-day assays were lower than 14.2%, while the trueness values were up to 119.2%. In addition, the device developed in this work is inexpensive when compared to commercial devices. In fact, the average cost of a PVOH/pectin hydrogel disk is about $0.15 per disk, taking in to account only the cost of the polymers, since the polycarbonate holder is reusable.

### 2.4. Analysis of Real Samples

Finally, the hydrogel-SPE device was applied for the determination of hormones in surface waters. The Belém River passes through the central region of Curitiba, which has a high population density and receives both domestic sewage and solid waste produced in this region. Estrone, 17β-estradiol and 17α-ethynylestradiol were quantified at concentrations of 1.11 to 1.58 μg L^−1^, while testosterone reached 3.58 to 5.84 μg L^−1^, which were the highest concentrations among all the studied hormones. Progesterone could also be identified in all samples, but at concentrations below its LOQ. Previous studies have shown a similar concentration for hormones in the same region. For example, Ide et al. [28] detected E2 and EE2 at concentrations of 1.42 and 1.48 μg L^−1^, respectively. These results prove that the developed SPE device based on hydrogel disks can be successfully used to extract steroidal hormones from water samples. 

## 3. Materials and Methods

### 3.1. Chemicals and Supplies

The hormone standards (>98% purity) used were 17α-ethynylestradiol (EE2), estriol (E3), estrone (E1), progesterone (PRO), testosterone (TES) (Fluka), and 17β-estradiol (E2) (Sigma-Aldrich, Saint Louis, MI, USA). Bisphenol Ad16 (BPAd_16_) (99.9%, Supelco, Bellefonte, PA, USA) was used as an internal standard. Ethyl acetate and methanol (J. T. Baker, HPLC grade) were used for the extraction procedure and chromatographic analysis. Ultrapure water was obtained from a Milli-Q system controlled at 18.2 MΩ cm (Millipore, São Paulo, SP, Brazil). Single stock solutions for each hormone and bisphenol Ad16 with concentrations of 1000 and 400 mg L^−1^, respectively, were prepared in methanol. Aqueous samples containing all hormones and the internal standard were prepared daily by spiking the stock solution with ultrapure water.

The reagents used for the derivatization step, N-methyl-trimethylsilyltrifluoroacetamide (MSTFA) and trimethylsilylimidazole (TMSI), were purchased from Sigma-Aldrich. The polymers used for the hydrogels were PVOH (MW 89,000–98,000, >99% hydrolyzed), purchased from Sigma-Andrich, and a commercial citrus pectin (PC) obtained from the Municipal Market of Curitiba (Curitiba, Brazil). The PC was purified by dialyzing against distilled water through a 12–14 kDa MW cut-off (Spectra/Por Cellulose Ester) for 72 h, followed by lyophilization. The dialyzed pectin is a high methoxyl pectin, as reported elsewhere [14]. Citric acid (Qhemis) was used as the crosslinking agent.

### 3.2. Preparation of the Hydrogel Disks 

First, aqueous dispersions of PVOH (5%, 10%, 15% and 20%, *w*/*v*) and dialyzed PC (1%, 2%, 3% and 4%, *w*/*v*) were prepared using ultrapure water. Then, the PVOH and pectin dispersions were mixed in a 1:1 (wt.) proportion. Table S1 (Supplementary Material) shows the initial concentrations of the dispersions employed in the preparation of the hydrogel disks studied. The resultant dispersion was stirred at room temperature until complete mixing of the polymers. Next, citric acid, used as a crosslinking agent, was added under agitation into the dispersion. In this step, a final concentration of 10% with respect to the total weight of the polymers was reached. Finally, 1 g of the dispersion was cast in a 10 mL beaker, used as a template, and left in an oven at 60 °C until completely dry. Then, the dried hydrogel was cut into disks with a stainless-steel blade. Hydrogels employing only PVOH or only pectin were prepared according to the same above mentioned procedure.

### 3.3. Physical-Chemical Characterization of the Hydrogel

The swelling index (SI) of the hydrogels was determined according to Equation (1) as follows:

(2) $$\mathrm{SI}(\%)\;=\;\frac{W_{f}\;-\;W_{i}}{W_{i}}100$$

The dried gels were weighed (W_i_) and immersed in 30 mL of ultrapure water. Then, the mass of the swollen gels (W_f_) was monitored for 300 min, when a constant mass was reached. Before weighing, in order to remove excess fluid, the hydrogels were slightly pressed against an absorbing paper.

The hydrogel water loss percentage (syneresis) was measured at room temperature (25 °C) and in a refrigerator (4 °C). First, the gels were immersed in ultrapure water for 90 min and slightly pressed against an absorbing paper. The gels were weighed at their equilibrium swelling (W_eq_) and after every 30 min interval (W_t_) for 300 min. The water loss percentage was calculated by Equation (2) as follows:

(2) $$\mathrm{Water}\;\mathrm{loss}(\%)\;=\;\frac{W_{\mathrm{eq}}\;-\;W_{t}}{W_{\mathrm{eq}}}100$$

### 3.4. Scanning Electron Microscopy (SEM) Analysis

The dry hydrogels were frozen in liquid nitrogen and fractured. Images of the fractured cross-section were obtained after metallization with gold (Balzers Union sputter-coater, model SCD 030). The scanning electron microscope was a JOEL^,^ JSM-6360LV model, operated at 10 kV.

### 3.5. Hydrogel-SPE Procedure

**Figure 5:**
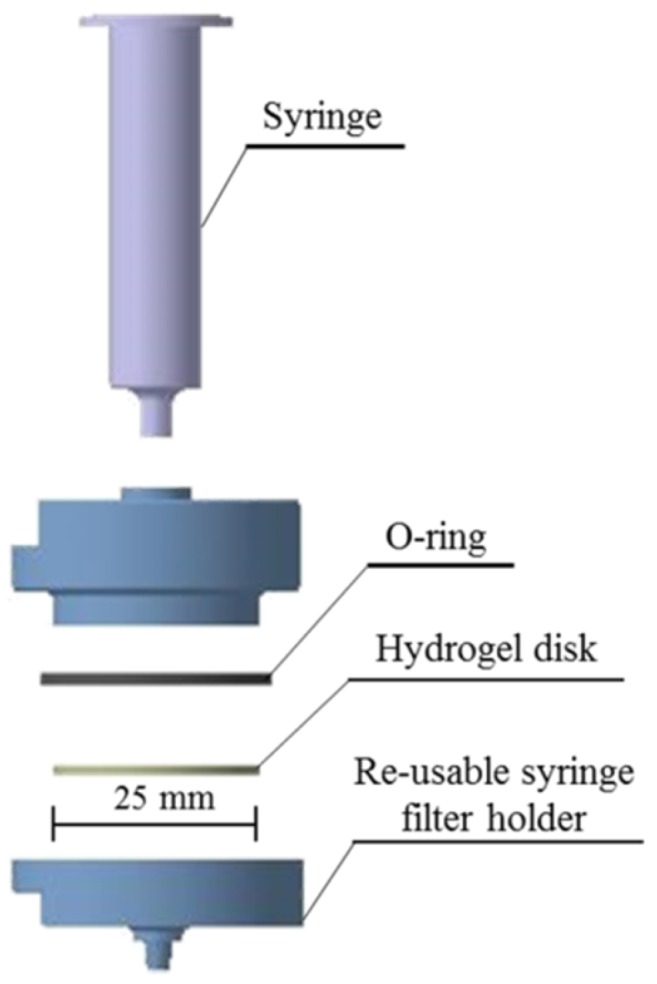
Schematic of the hydrogel-SPE device.

The hydrogel-SPE assembly consisted of a polycarbonate reusable syringe filter holder, which is where the hydrogel disk was placed. A syringe was attached to the holder without a plunger through which the sample was discharged (Figure 5).

Before the extraction, each dried hydrogel disk was positioned inside a reusable polycarbonate syringe filter holder (Sartorius) with a 25 mm diameter and hydrated with 10 mL of ultrapure water. Aqueous samples were passed through the hydrogel disk using a vacuum manifold (model Visiprep, Supelco, Bellefonte, PA, USA). Then, the disk was air-dried under a vacuum, and the elution of the analytes was carried out at a flow rate of 1 mL min^−1^. Finally, the extracts were completely dried and reconstituted with 200 µL of methanol and carried on to the derivatization procedure.

To evaluate the better extraction conditions, a two-level full factorial design with three factors (2^3^) was performed. The optimized parameters were the following: pH (4.5, 6.0, and 7.5), sample volume (100, 150, and 200 mL), and flow rate (2, 3, and 4 mL min^−1^). The peak area values were used has response. Factorial design calculations and Pareto charts were performed by Statistica software (Mathworks, Natick, MA, USA, version 7.0.1).

Moreover, the optimization process of the elution step was carried out taking into consideration the number of elutions (1, 2, 3, 4, 5 and 6 elutions) of 1.0 mL of methanol. The optimization process was accomplished in triplicate.

### 3.6. Derivatization Step and Gas Chromatography-Tandem Mass Spectrometry (GC-MS/MS) Analysis

For the derivatization step, 100 µL of the methanolic extracts were completely dried, 30 µL of MSTFA:TMSI (99:1, *v*/*v*) were added to the samples, and the silylation reaction was carried out in the same oven at 70 °C for 30 min. Then, 70 µL of ethyl acetate were added to the samples and 1 µL was injected into the GC-MS/MS system.

The GC-MS/MS analysis was carried out on a Shimadzu system consisting of a GC2010 Plus gas chromatograph hyphenated to a TQ8040 triple quadrupole mass spectrometer (Shimadzu, Kyoto, Japan). The GC was equipped with an AOC5000 auto-injector and a split-splitless injector. The chromatographic separation occurred in a GC column SH-RTX-5 ms (30 m × 0.25 mm × 0.25 µm) with helium 5.0 as the carrier gas at a flow rate of 1.0 mL min^−1^. A sample volume of 1 µL was injected in a split ratio of 1:10. Both the injector and ion source temperatures were set at 250 °C. The interface temperature was 300 °C. The GC oven temperature was kept at 220 °C for 2 min, followed by 20 °C min^−1^ to 280 °C (1 min), 2 °C min^−1^ to 290 °C and 20 °C min^−1^ to 300 °C (1.5 min). The total analysis time was 13 min. The triple quadrupole mass spectrometer was operated in multiple reaction monitoring (MRM) mode with electron ionization at 70 eV. For each analyte, one quantification and two confirmation transitions were determined (Table S2—Supplementary Material).

Although the goal of this paper was not to describe a validated analytical method, the feasibility of the application of the developed device for quantitative analysis was evaluated. For this purpose, the steroidal hormones were chosen as target analytes, and some merit parameters were studied, such as limit of quantification (LOQ), RSD, accuracy, linear range, and correlation coefficient (r). Moreover, to evaluate the reuse, and consequently, the robustness of the hydrogel disks, ten subsequent extractions with the same disk were performed under the same extraction and analysis conditions described previously. Between the extractions, the material was cleaned with methanol and ultrapure water, avoiding the memory effect.

### 3.7. Surface Water Samples

Surface water samples were collected at three locations along the Belém River, in the city of Curitiba, Brazil. The sampling was carried out using 4 L amber glass bottles previously cleaned and rinsed with the sample. All water samples were filtered through a 0.6 µm fiberglass membrane (Macherey-Nagel, Düren, Germany), stored in amber glass bottles at 4 °C for a maximum period of 24 h and analyzed according to the developed method. Before the extraction, the surface water samples were spiked with the IS (BPA-d_16_), resulting in a concentration of 25.0 ng mL^−1^.

## 4. Conclusions

In this work, an amphiphilic, reproducible, free of memory effects, and cost-effective sorbent phase was obtained. The use of hydrogels as the sorbent phase showed potential for the extraction of media and high-polarity compounds from aqueous matrices, which has been a challenge for extraction techniques in general. The evaluation of the physical-chemical properties of the polyvinyl alcohol and pectin (a natural polymer) hydrogel proved that the water content within the hydrogel matrix and its functional groups are very important to ensure satisfactory polar interactions with the analytes.

Compared with previous studies on SPE/GC-MS for the analysis of organic contaminants in water, similar precisions and accuracies were achieved with the device developed in this work, but the PVOH/pectin extraction phase was more stable than commercial SPE phases, allowing for reutilization of the extraction device. However, further studies are necessary to reach LOQ values as low as those obtained in SPE when using commercial extraction phases. On the other hand, the new hydrogel-based material developed was successfully used to determine steroidal hormones in surface waters. Therefore, using an easily prepared hydrogel (in disk form) can be an alternative for extraction techniques, especially due to its characteristics of modulating and containing several polar groups in its structure, as well as the simplicity of its preparation and its low cost.

## Figures and Tables

**Table 1:** Precision and accuracy of the hydrogel-solid phase extraction (SPE) procedure.

Analytes	Concentration Evaluated/µg L^−1^	Intra-Assay Precision (*n* = 3)			Concentration Evaluated/µg L^−1^	Inter-Assay Precision (*n* = 3)		
		Measured Conc./µg L^−1^	RSD/%	Accuracy/%		Measured Conc./µg L^−1^	RSD/%	Accuracy/%
E1	40.0	38.6 ± 0.21	0.5	96.7	50.0	50.3 ± 2.9	5.7	100.5
	80.0	71.8 ± 2.9	4.1	89.7	100.0	102.6 ± 1.9	1.9	102.6
E2	40.0	33.1 ± 1.5	1.9	82.6	50.0	50.5 ± 3.1	6.2	100.9
	80.0	63.9 ± 1.5	2.4	80.0	100.0	99.8 ± 8.29	8.3	99.8
TES	40.0	38.1 ± 4.0	10.4	95.4	50.0	50.3 ± 3.0	6.0	100.6
	80.0	72.8 ± 5.2	7.2	91.0	100.0	98.7± 3.8	3.8	98.7
EE2	40.0	37.6 ± 2.0	5.4	94.0	50.0	46.9 ± 2.8	6.0	93.7
	80.0	85.1 ± 7.6	8.9	106.3	100.0	100.8 ± 4.8	4.7	100.8
PRO	40.0	35.6 ± 4.1	11.5	88.9	50.0	49.1 ± 10.9	22.2	98.1
	80.0	83.7 ± 10.3	12.3	104.7	100.0	102.4 ± 6.8	6.6	102.4
E3	40.0	40.4 ± 3.0	7.3	101.0	50.0	49.9 ± 1.7	3.4	99.7
	80.0	85.0 ± 6.5	7.6	106.2	100.0	108,2 ± 8.6	7.9	108.2

## Supplementary Materials

Figure S1: Pareto charts of the effects of pH, sample volume (V), and flow rate (F) on the extraction efficiency. Table S1: Hydrogel disk composition. Table S2: Optimized parameters for tandem MS.
