# Supplementary material for: Evaluation of Polyvinyl Alcohol/Pectin-Based Hydrogel Disks as Extraction Phase for Determination of Steroidal Hormones in Aqueous Samples by GC-MS/MS
Source: Molecules. 2018 Dec 22;24(1):40. doi: 10.3390/molecules24010040 (PMC6337582; doi:10.3390/molecules24010040)
Supplement: Supplementary file 1 [file molecules-24-00040-s001.pdf]

SUPPLEMENTARY MATERIAL

Figure S1. Pareto charts of effects of pH, sample volume (V), and flow rate (F) on the extraction efficiency.

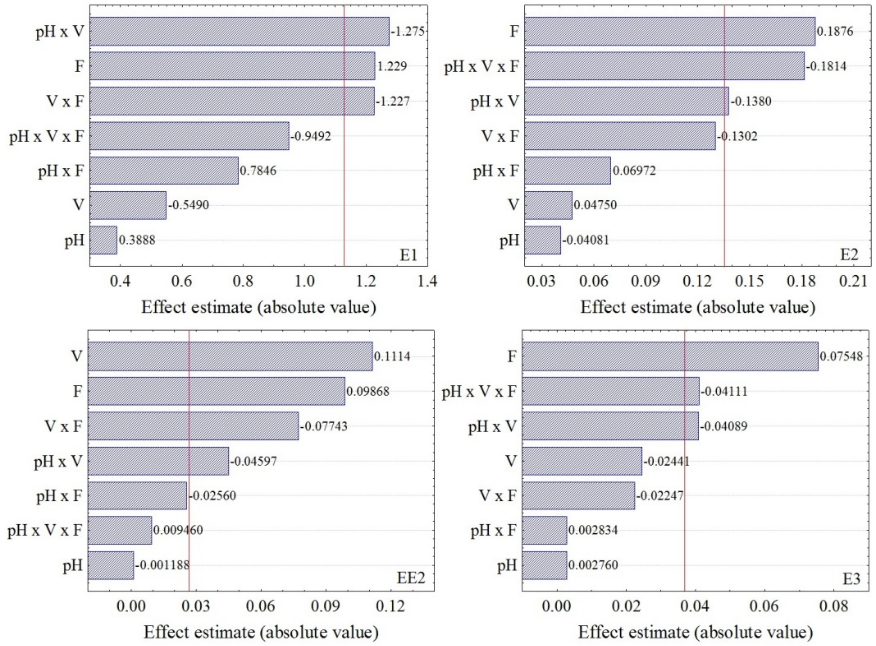

Table S1. Hydrogel disk composition.

Dispersion	Disk							
	a	b	c	d	e	f	g	h
PVOH/ %(w/v)	5	10	15	20	5	10	10	15
Pectin/ %(w/v)	1	2	2	4	2	1	3	2

a. P5PC1; b. P10PC2; c. P15PC3; d. P20PC4; e. P5PC2; f. P10PC1; g. P10PC3; h. P15PC2, were P = PVOH and PC = pectin.

Table S2. Optimized parameters for tandem MS.

Compound	Retention time/min	Quantification transition (CE/eV)	Confirmation transitions (CE/eV)
BPA - d16	4.58	368 > 73 (25)	368 > 197 (22)
			368 > 296 (25)
E1	7.18	342 > 257 (13)	342 > 244 (16)
			342 > 73 (28)
E2	7.38	285 > 73 (28)	285 > 205 (19)
			285 > 229 (19)
TES	7.60	129 > 73 (10)	129 > 58 (28)
			129 > 127 (13)
EE2	8.31	425 > 193 (19)	425 > 167 (19)
			425 > 231 (25)
PRO	8.91	124 > 109 (13)	124 > 96 (10)
			124 > 81 (22)
E3	9.12	311 > 255 (13)	311 > 73 (25)
			311 > 282 (19)

CE: collision energy.
